# Children with problem-drinking parents in a Swedish national sample: is the risk of harm related to the severity of parental problem drinking?

**DOI:** 10.1093/eurpub/ckad022

**Published:** 2023-02-15

**Authors:** Mats Ramstedt, Jonas Raninen, Peter Larm, Michael Livingston

**Affiliations:** Department of Clinical Neuroscience, Karolinska Institutet, Stockholm, Sweden; The Swedish Council for Information on Alcohol and Other Drugs (CAN), Stockholm, Sweden; Department of Public Health Sciences, Stockholm University, Stockholm, Sweden; Department of Clinical Neuroscience, Karolinska Institutet, Stockholm, Sweden; The Swedish Council for Information on Alcohol and Other Drugs (CAN), Stockholm, Sweden; Centre for Alcohol Policy Research, La Trobe University, Melbourne, Australia; Department of Public Health Sciences, Stockholm University, Stockholm, Sweden; Department of Clinical Neuroscience, Karolinska Institutet, Stockholm, Sweden; Centre for Alcohol Policy Research, La Trobe University, Melbourne, Australia; National Drug Research Institute, Curtin University, Perth, Australia

## Abstract

**Background:**

The aim of this paper is to examine the link between severity in exposure to parental problem drinking in a Swedish national population sample of children aged 15–16 years. Specifically, we assessed whether the risk of poor health, poor relationships and a problematic school situation increase with severity in exposure to parental problem drinking.

**Methods:**

National population survey from 2017 with a representative sample of 5 576 adolescents born in 2001. Logistic regression models were used to estimate odds ratios (ORs) with 95% confidence intervals (95% CIs). A short version of The Children of Alcoholics Screening Test, CAST-6, was used to identify children with problem-drinking parents. Health status, social relations and school situation were assessed by well-established measures.

**Results:**

The risk of having poor health, poor school performance and poor social relations increased with severity of parental problem drinking. The risk was lowest among children least severely affected (Crude models ranged from OR: 1.2, 95% CI 1.0–1.4 to OR: 2.2, 95% CI 1.8–2.6) and highest among children most severely affected (Crude models ranges from OR: 1.7, 95% CI 1.3–2.1 to OR: 6.6, 95% CI 5.1–8.6). The risk became lower when adjusting for gender and socioeconomic position but were still higher compared to children without problem-drinking parents.

**Conclusions:**

Appropriate screening and intervention programs are necessary for children with problem-drinking parents especially when exposure is severe but also at mild forms of exposure.

## Introduction

A number of studies have shown that children of problem-drinking parents have an elevated risk of adverse consequences in many areas, e.g. health problems, bad family and peer relations, substance use problems and poor school adjustment.^1–8^ Impaired parenting and social strains associated with having a problem-drinking parents are suggested mechanisms behind these results but also that parental alcohol problems tend to co-vary with other risk factors such as limited socioeconomic resources.^9^ Although it is well established that parental alcohol problems have adverse effects on the children, there is a shortage of research on how the severity in exposure to parental alcohol problems is related to the risk of adverse outcomes in children. Such knowledge is important for policy makers when they are deciding who should be the target of interventions; whether it should be the smaller group severely affected by parental drinking or also include the larger group of children with a milder form of exposure to parental drinking.

A study by Raitasalo *et al*.^10^ is to our knowledge, the only study addressing this question and they hypothesized that the more severe parental alcohol problems, the greater the risk of negative outcomes for their children. However, using a retrospective population-based cohort study, based on health care and social welfare registers, they found that variations in severity in parental alcohol problems were not associated with the risk of harm for children. This result was explained by the high severity of harm among all cases ending up in registers and that it is likely that ‘a threshold’ for these risks is realized on lower levels of alcohol problems not captured with register data. To assess if there is such a threshold, they called for an analysis of severity in a population sample also including parents with milder forms of alcohol problems as well as parents with more severe problems not ending up in care. The latter group is important to take into account since previous studies suggest that only 10–20% of parents with an alcohol use disorder seeks treatment.^11^

The aim of this article is therefore to assess the importance of severity in exposure to parental problem drinking in a Swedish national population sample of children aged 15–16 years. More specifically, the number of problems experienced with parental drinking in the measure CAST-6 (Children of alcoholics screening test) will be analysed in relation to the risk of poor health, poor relationships and a problematic school situation. A recent study based on this data found that children experiencing at least three of the six problems included in CAST-6 (the original recommended cut-off) had an elevated risk of poor general health as well psychosomatic problems compared with other children.^12^ These children were also more likely to use medication for depression, sleeping difficulties and anxiety. Furthermore, their social relations were worse compared with other children, especially with their father, and they had more problems at school. Similar findings have been found in other studies based on national samples of children.^9^^,^^13^^,^^14^

However, no previous study has examined if the risk of problems is influenced by severity in parental problem drinking. As girls and children with a lower socioeconomic position experience more problems related to parental drinking as well as more health problems^12^^,^^15^^,^^16^ gender and socioeconomic position will also be adjusted for in the analyses.

The research question to be addressed in this article is the following:

- To what extent does the risk of poor health, poor relationships and a problematic school situation increase with severity in exposure to parental problem drinking in a Swedish population sample of children aged 15–16 years?

## Methods

We used baseline data from Futura01, which is a national prospective longitudinal study of ∼5549 Swedish youth born in 2001. The baseline data collection (T1) was carried out during spring 2017 when the respondents attended the 9th grade and were 15–16 years old. The sampling was conducted by Statistics Sweden and includes a random sample of 500 schools. In each school one class was selected randomly and all students in the selected classes were then asked to participate in the study by filling out a paper and pen questionnaire during school hours. After several reminders 343 schools decided to participate, implying a participation rate of 68.8% at the school level. All in all, 6777 questionnaires were returned of which 5576 had filled in the questionnaire and given informed consent to participate in the study. This corresponds to a response-rate of 82.3% at the individual level. Nineteen observations were excluded due to missing answers on individual items and eight respondents had given unrealistic answers. Seventy-two respondents did not answer CAST-6 resulting in 5477 complete cases in the analytical sample. Analysis of the representativeness of the sample showed no significant differences between schools that participated and those that did not. At the school level, meta-data on parental education and immigrant background was compared as was the overall average grades for all the year 9 students. The schools also had a high geographical representation and similar prevalence rates as in other national school surveys conducted at the same time but without demanding informed consent. Nevertheless, there was a minimal overrepresentation of boys in the current sample (49.5%) in comparison with the population (49%).^17^

### Measures

A short version of The Children of Alcoholics Screening Test, CAST-6, was used to discriminate between children with problem-drinking parents and other children. CAST-6 includes 6 true/false items instead of the 30 items from the original scale and has been shown to have the same validity.^18^ The six questions concern four areas; perceptions of parental alcohol problems, attempts to control parental drinking, perceptions of marital discord and efforts to escape adverse consequences. Children responding ‘true’ to at least three statements are traditionally defined as having a problem-drinking parents although two positive responses have also been suggested.^18^^,^^19^

In this article, we will use CAST-6 as an ordinal scale of severity with the assumption that more reported problems indicate a more severe exposure to parental problem drinking. In order to obtain enough cases within each category, we collapsed the responses to CAST-6 as follows: 1–2 problems (low severity), 3–4 (moderate severity) and 5–6 (high severity). We then examined whether the prevalence of key problems varied across these three levels of severity. The following problems were analysed: poor physical and mental health, poor social relationships and poor school satisfaction. To estimate risks for these adverse consequences, the outcomes were dichotomized into a negative and positive category. How these outcomes were measured specifically are described below.

*Physical and mental health* include the general health status and three indicators of psychosomatic problems including stomach pain, stress and headache.

- *General health status* was measured with a five-point Likert scale ranging from ‘very good’, ‘rather good’, ‘either good or bad’, ‘rather bad’ or ‘very bad’. Respondents were classified as having poor health status if they reported ‘very bad’, ‘rather bad’ or ‘neither good or bad’.
- *Psycho-somatic problems* were measured by questions adapted from the Health Behaviors of School age Children (HBSC) including how often they suffered from stomach pain, stress and headache. The responses were dichotomized into at least ‘once a week’ and less often based on five response options ranging from ‘everyday’ to ‘seldom/never’.

The quality of the respondents’ *social relations* was measured with questions of how satisfied the respondent was with his/her relationship to father, mother and friends ranging from very happy, happy, not so happy to not happy at all. Response options ‘not so happy’ and ‘not happy at all’ were assumed to measure a poor relationship.

To measure *school satisfaction,* we used the question ‘How do you like school?’ and responses with a five-point Likert scale ranging from ‘very good’, ‘rather good’, ‘neither good or bad’, rather bad or very bad. Low school satisfaction was defined as ‘neither good or bad’, ‘rather bad’ or ‘very bad’. Another aspect of school satisfaction was *truancy* which was measured by the question ‘Do you skip school?’ with those responding at least ‘some time every semester’ being positive cases. Finally, experiences of having been bullied were measured using questions addressing whether this had happened during the last 12 months.

The analyses consist of a graphical presentation of the proportion with various problems at different numbers of problems (0–6) with parental drinking. Estimation of logistic regression models was used to compare the risk of problems at low, moderate and high severity in exposure to parental problem drinking to those without exposure to parental problem drinking. Crude models as well as models adjusted for gender and socioeconomic position were estimated. As an indicator of low socioeconomic position, we used a measure of not being able to buy things others can afford based on the question: *Think back to the last 12 months. Have you ever been unable to buy something that you wanted and that other’s your age have, because you could not afford it?* Those responding ‘yes’ to this question were assumed to have a low socioeconomic position. The analysis was not pre-registered and the results should be considered exploratory.

## Results

### Health status

**Figure 1:**
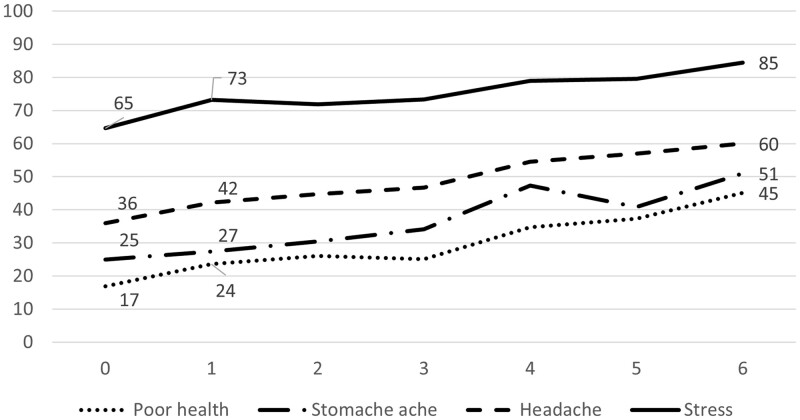
Proportion of children reporting different health problems (*Y*-axis) by number of problems with parental drinking according to CAST-6 (*X*-axis)

The proportion of adolescents reporting a poor health status increased by the number of reported problems with parental drinking according to all studied health indicators, i.e. poor general health and experiences of psychosomatic problems such as stomachache, headache and stress (figure 1). The risk increased already for children experiencing one problem compared with children not having problem-drinking parents. The highest risk is found in the group experiencing the highest number of problems, i.e. six harms.

The increased risk of poor health status by severity in exposure to parental drinking is verified in table 1 where findings from logistic regressions are shown. It is demonstrated that even a mild exposure (one or two harms) is associated with an elevated risk that is statistically significant (odds ratios (ORs) of 1.2–1.6, *P* < 0.01). This pattern is found for all health indicators and remains with control for gender and socioeconomic position in the adjusted models, except for the risk of stomachache.

**Table 1:** Risk of various adverse consequences among children with low, moderate and high severity in exposure to parental problem drinking according to a division of CAST-6 into three categories

Outcome	Low severity (*n* = 951)		Moderate severity (*n* = 422)		High severity (*n* = 297)	
	Crude	Adjusted	Crude	Adjusted	Crude	Adjusted
Health status						
Poor health	1.6 [1.3–1.9]	1.5 [1.2–1.8]	2.1 [1.6–2.6]	2.0 [1.5–2.5]	3.6 [2.8–4.7]	3.1 [2.3–4.1]
Stomachache	1.2 [1.0–1.4]	1.1 [0.9–1.3]	2.0 [1.6–2.5]	1.8 [1.5–2.3]	2.7 [2.1–3.4]	2.0 [1.5–2.6]
Headache	1.3 [1.2–1.5]	1.3 [1.1–1.5]	1.8 [1.4–1.8]	1.6 [1.3–2.0]	2.6 [2.0–3.3]	2.0 [1.6–2.6]
Feelings of stress	1.5 [1.2–1.7]	1.4 [1.1–1.6]	1.7 [1.3–2.7]	1.6 [1.2–2.0]	2.6 [1.9–3.6]	2.0 [1.4–2.8]
Social relations						
Poor relationship with father	2.2 [1.8–2.6]	1.9 [1.5–2.4]	4.1 [3.2–5.2]	3.7 [2.9–4.8]	6.6 [5.1–8.6]	5.0 [3.7–6.7]
Poor relationship with mother	1.9 [1.5–2.5]	1.8 [1.4–2.4]	2.7 [2.0–3.6]	2.5 [1.8–3.5]	3.5 [2.5–4.9]	2.8 [1.9–4.0]
Poor relationship with friends	1.4 [1.1–1.8]	1.2 [0.9–1.6]	1.9 [1.4–2.7]	1.6 [1.1–2.3]	2.2 [1.5–3.1]	2.0 [1.4–2.4]
School situation						
Have been bullied	1.8 [1.4–2.3]	1.7 [1.3–2.3]	3.0 [2.2–4.1]	2.8 [2.0–3.9]	3.8 [2.8–5.3]	3.6 [2.5–5.2]
Do not enjoy school	1.5 [1.3–1.8]	1.4 [1.1–1.6]	1.9 [1.5–2.4]	1.7 [1.3–2.1]	2.3 [1.8–3.0]	1.9 [1.4–2.5]
Have schooled	1.5 [1.3–1.7]	1.5 [1.2–1.7]	1.9 [1.5–2.4]	1.8 [1.5–2.3]	1.7 [1.3–2.2]	1.5 [1.19–2.0]

Odds ratios estimated in logistic regressions with children reporting no parental problem drinking as a reference group (confidence intervals in parenthesis).

The risk becomes higher in groups with moderate and high severity in exposure to parental drinking respectively where all estimates are statistically significant also in adjusted models.

### Social relationships

**Figure 2:**
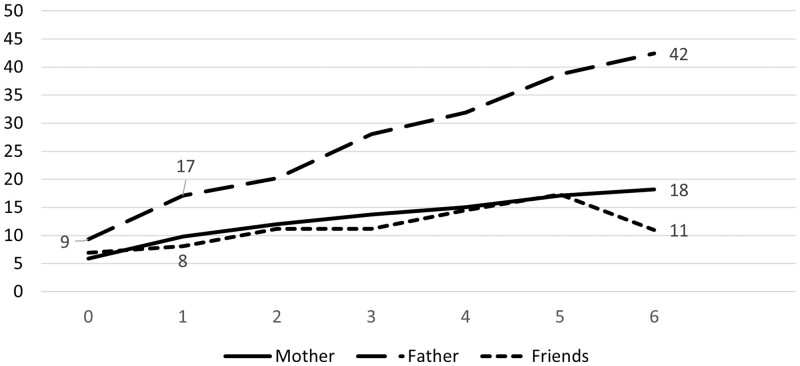
Proportion of children reporting having a poor relationship with parents and friends (*Y*-axis) by number of problems with parental drinking according to CAST-6 (*X*-axis)

The number of reported problems with parental drinking is associated with a worse relationship with friends and both parents (figure 2). The association is especially steep for the relation to the father which sharply becomes poorer with an increasing number of experienced problems with parental drinking.

The graphical result is reflected in logistic regressions with ORs for a poor relation to the father being higher at all problem levels in comparison with the relation to the mother and friends (table 1). For instance, among children being most severely affected by parental drinking (5–6 problems), the OR for a poor relation with the father is 6.6 compared with 3.5 for the mother and 2.2 for friends (crude models). Corresponding figures for mild exposure are 2.2, 1.9 and 1.4. These estimates became somewhat weaker in adjusted models but were still statistically significant.

### School situation

**Figure 3:**
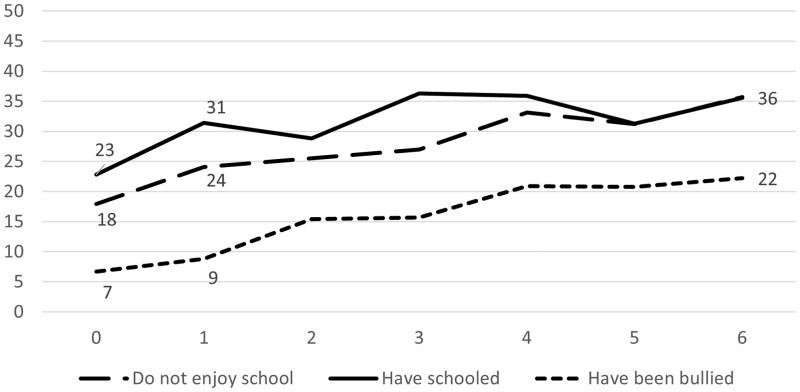
Proportion of children reporting a poor situation in school (*Y*-axis) by number of problems with parental drinking according to CAST-6 (*X*-axis)

The proportion of adolescents reporting a poor *school situation* increases by the number of reported problems with parental drinking in a similar manner according to all three studied indicators (figure 3).

The increased risk of a poor school situation by severity in exposure to parental drinking is verified in table 1 where it is also demonstrated that a low severity is associated with an elevated risk. Moreover, the risk becomes higher at moderate exposure and highest among the most severely exposed. This pattern is found for each indicator of a poor school situation, i.e. for the risk of not enjoying school, to have schooled and to have been bullied. All estimates were statistically significant when controlling for gender and socioeconomic position.

## Discussion

Although it is well established that children with problem-drinking parents have an elevated risk of a poor life situation in many respects, there is a lack of studies addressing the importance of severity in exposure to parental problem drinking. We found only one study where there was no evidence that severity was related to the risk of harm in children.^10^ However, this study was based on a clinical sample where it was assumed that a threshold for a high risk already had been exceeded. Therefore, the aim of this article was to address this question on a national population sample assumed to include milder forms of exposure to parental problem drinking as well as more severe exposure to parental problem drinking not reached by the treatment system.

We found that higher severity in exposure to parental problem drinking among 15–16-year-olds in a Swedish national sample was related to a higher risk that the child had a reduced life situation in terms of poor health, poor social relations and poor school performance. An elevated risk was found already at low severity in exposure suggesting that there is no clear threshold for when the risk of problems becomes elevated. This finding has some support in earlier studies suggesting that also low levels of parental drinking may be associated with increased risk of psychosomatic problems to children.^20^ That a milder form of exposure is associated with an elevated risk of harm in a national population sample is important knowledge since this group is more numerous than clinical populations. Thus, although the risk is lower at the individual level, this group account for a relatively large proportion of the total number harmed from parental problem drinking in the population. Still, children with the most severe exposure (reporting five or six problems) were consistently at the highest risk of a poor life situation according to all indicators included in this study. This was most obvious for having a poor relationship with the father where 40% of those with the most severe exposure to parental drinking had a bad relationship compared with 9% among other children. The estimated OR was subsequently also highest (6.6) and significant also with control for gender and socioeconomic position. While our data did not contain information of which one of the parents drinking were problematic to the child, this result is in line with previous research. For instance, on the basis of data from a Swedish general population sample of adults, Sundin^21^ showed that 82.8% of those who grew up with a problem-drinking parents reported that it was the father compared with 26.5% who mentioned the mother. This result is compatible with the well-known fact that men drink more alcohol than women^22^ and suggests that fathers drinking account for the majority of harm to children from parental drinking. Other harms that were especially elevated for this severely affected group were a poor general health and exposure to bullying. Thus, there is evidence of a clustering of many different problems implying that children who are severely affected by parental drinking is an especially vulnerable group.

### Limitations

There are several limitations in this study that need to be acknowledged. First, causality cannot be established here due to the cross-sectional design. Thus, we cannot rule out that some other factor is related to both exposure to parental problem drinking, and the included measures of a poor life situation. Although it is reassuring that the findings remained with control for socioeconomic position and gender, we were not able to control for the suggested high comorbidity between mental disorders and alcohol use disorders.^23^ Thus, mental illness among parents may have increased the risk for problems in these children in addition to parental alcohol consumption. Still, it should be noted that these factors need to be related to the risk of harm in a similar manner as the measure of exposure, i.e. the number of problems included in CAST-6, in order to explain the observed association between severity in parental drinking and risk of harm. The lack of temporality may also increase the risk of reverse causality. However, although it cannot be excluded, it seems unlikely that the perception of parents’ alcohol use as problematic should be a result of health problems, poor social relationships or problems in school among adolescents. Another limitation is that exposure to problem-drinking parents according to CAST-6, is based on self-reported information from children and thus their perceived experience of problems related to their parents drinking. Thus, there is no objective information on whether parental drinking actually is problematic or would be defined as such in a clinical setting. Still, it can be argued that if children perceive negative effects, this is an important marker for defining parents drinking as problematic, independent of whether the drinking pattern would qualify for risk drinking or alcohol dependence. Another limitation is that CAST-6 refers to lifetime experiences of parental problem drinking whereas the problem outcomes refer to the last 12 months. This means that some parents defined as problem-drinking parents may have stopped or reduced their drinking and that current parental drinking has no or little adverse impact on the child. It is worth noting in this context however that studies show that problematic parental drinking during adolescence may have longstanding effects and influence the child also when the drinking problem has stopped.^24^ Finally, we do not know whether CAST-6 performs differently when parents are separated and where the child have less contact to one of their parents. It is possible that the scale in such cases underestimates the harms, since the questions, implicitly, basically assume that they live together.

### Strengths

The only previous study on the importance of severity of parental alcohol problems in relation to the effects for children was based on the most more severe clinical cases of parental alcohol problems with limited generalizability to the wider population of adolescents. The major strength of this study is thus that it used a large and high-quality national sample of young people with a high representativeness.^17^ Furthermore, the analysis was based on established and validated measures of both parental problem drinking and various adverse outcomes.

## Conclusion

The risk of problems related to health, social relations and school performance increased with severity of exposure to parental problem drinking for children in a national sample of 15–16-year-olds in Sweden. An elevated risk was found also at low severity in exposure to parental problem drinking and became higher by increasing severity. These findings suggest that children with problem-drinking parents should be offered help especially at severe exposure to parental problem drinking but that support is warranted also at milder forms of exposure.

## Supplementary Material

ckad022_Supplementary_Data

## Data Availability

Data available on request.
