# Supplementary material for: Children with problem-drinking parents in a Swedish national sample: is the risk of harm related to the severity of parental problem drinking?
Source: Eur J Public Health. 2023 Feb 15;33(2):312–6. doi: 10.1093/eurpub/ckad022 (PMC10066494; doi:10.1093/eurpub/ckad022)
Supplement: ckad022_Supplementary_Data [file ckad022_supplementary_data.docx]

Table A. Descriptive information for the included indicators of harm (%)

| Harm indicator |  |  |  |  |  |
| --- | --- | --- | --- | --- | --- |
| *How is your health?* | Very good | Good | Either good or bad | Bad | Very bad |
|  | 31 | 48 | 15 | 5 | 1 |
|  |  |  |  |  |  |
| *How often last 6 months did you have?* | Everyday | Few times a week | Once a week | Sometimes per month | Seldom |
| Stomachache | 2 | 12 | 13 | 39 | 33 |
| Headache | 5 | 17 | 17 | 33 | 28 |
| Stress | 15 | 32 | 20 | 23 | 9 |
| *How satisfied are you with your relationship with:* | Very happy | Happy | Not so happy | Not happy at all | Not relevant/do not know |
| Mother | 65 | 26 | 6 | 2 | 1 |
| Father | 55 | 28 | 9 | 5 | 3 |
| Friends | 54 | 37 | 6 | 2 | 1 |
| *How do you like school?* | Very good | Good | Either good or bad | Bad | Very bad |
|  | 34 | 46 | 15 | 4 | 2 |
| *Do you skip school?* | No | Sometime per semester | 1-3 /month | Once a week | Several times a week |
|  | 73 | 16 | 7 | 2 | 2 |
| *Have you been bullied?* | No | Yes | Don’t know | - | - |
|  | 83 | 8 | 9 | - | - |
